# Supplementary material for: Venomix: a simple bioinformatic pipeline for identifying and characterizing toxin gene candidates from transcriptomic data
Source: PeerJ. 2018 Jul 31;6:e5361. doi: 10.7717/peerj.5361 (PMC6074769; doi:10.7717/peerj.5361)
Supplement: Supplemental Information 3 [file peerj-06-5361-s003.gz › FinalOutput_E-20/Snaclec_rhodocetin_subunit_delta_1/finaltree.pdf]

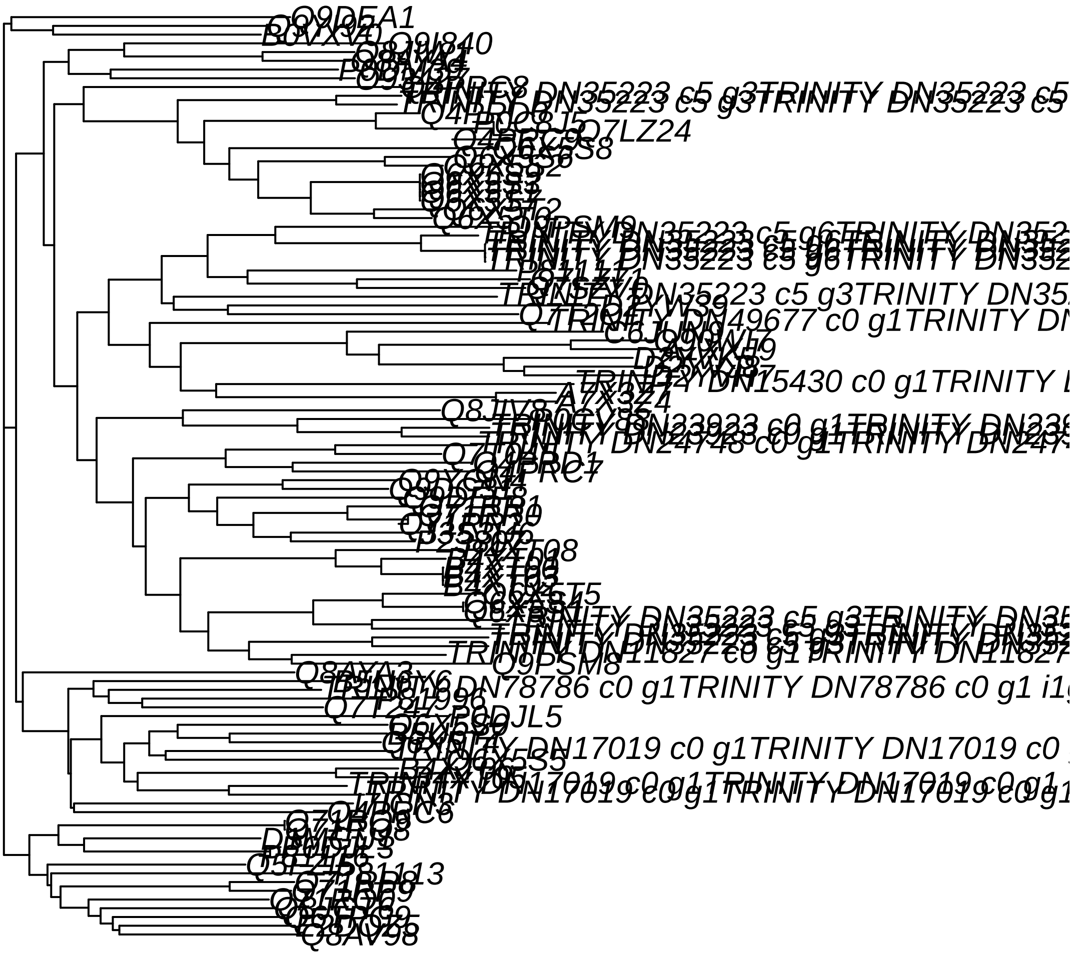

Q9DEA1
B0VXV0
Q9I840
Q7LZ24
TRINITY DN35223 c5 g3TRINITY DN35223 c5
TRINITY DN49677 c0 g1TRINITY DN
TRINITY DN15430 c0 g1TRINITY
A7X3Z4
TRINITY DN24748 c0 g1TRINITY DN247
Q4PRC7
Q9PSM8
TRINITY DN78786 c0 g1TRINITY DN78786 c0 g1 i1g
P0DJL5
TRINITY DN17019 c0 g1TRINITY DN17019 c0
TRINITY DN11827 c0 g1TRINITY DN11827
Q8AV98
